# Advancing Solar–Thermal–Electric Conversion via Phase-Change Materials: Current Status and Emerging Opportunities

**DOI:** 10.34133/research.1480

**Published:** 2026-11-12

**Authors:** Tao Huang, Pinggui Zhu, Bo Zhou, Lingfeng Ouyang, Mingtai Hou, Xingchao Han, Xiaoqiang Zhang, Jianjun Hou, Yang Si, Zeyi Jiang, Nien-Chu Lai, Cunhai Wang, Fuqiang Chu, Yulong Ding

**Affiliations:** ^1^ School of Energy and Electrical Engineering, Qinghai University, Xining, Qinghai, China.; ^2^ Birmingham Centre for Energy Storage, School of Chemical Engineering, University of Birmingham, Birmingham, UK.; ^3^ Global Energy Interconnection Research Institute Europe GmbH, Berlin, Germany.; ^4^ School of Energy and Environmental Engineering, University of Science and Technology Beijing, Beijing, China.

## Abstract

Phase-change materials (PCMs) offer an effective strategy for mitigating the intermittency of solar energy by regulating temporal temperature profiles through latent-heat storage. Their integration into solar–thermal–electric systems has enabled the synergistic coupling of solar-energy harvesting, thermal energy storage, and thermoelectric power generation. This field has evolved along 3 interconnected design dimensions: material composition and interfacial engineering, multiscale architectural design of composite PCMs, and system-level integration with thermoelectric generators (TEGs). At the material level, high-light-absorption and high-thermal-conductivity fillers combined with engineered interfaces have substantially improved photothermal conversion and heat transfer. At the structural level, porous aerogels, hierarchical thermal conduction networks, and biomimetic architectures have optimized light absorption and thermal transport while alleviating the trade-off between latent-heat storage and thermal conductivity. At the system level, PCM–TEG integration has extended power generation beyond daytime operation, with selected systems demonstrating continuous day–night generation. Despite these advances, large-scale deployment remains limited by high interfacial thermal resistance, insufficient cycling stability, and system complexity. Future progress will rely on physics-principle-driven design, molecular-scale interface engineering, adaptive phase-change systems, and physics-informed, artificial-intelligence-assisted multiscale multiphysics modeling to accelerate coordinated optimization and practical deployment.

## Introduction

Under the dual driving forces of global carbon neutrality initiatives and energy-structure transformation, the development of efficient and sustainable clean-energy technologies has become an important research focus in modern energy science [1]. Among various renewable energy resources, solar energy has attracted widespread attention owing to its abundance, environmental friendliness, and sustainability. In particular, solar-to-thermal conversion is regarded as one of the most promising solar-energy-utilization pathways because of its near-full-spectrum light-absorption capability and low energy-conversion losses. However, current solar–thermal technologies remain primarily limited to low-grade thermal utilization, making it difficult to achieve high-value energy output. Compared with conventional photovoltaic (PV) technologies, solar–thermal–electric (STE) conversion systems enable efficient, broadband utilization of solar energy through cascaded photon–phonon–electron energy-conversion processes while avoiding the intrinsic bandgap limitation of semiconductor photovoltaics [2]. Among them, thermoelectric generators (TEGs), based on the Seebeck effect, have demonstrated broad application prospects in remote power supply, freshwater production [3], flexible and wearable electronics [4], aerospace systems [5], anti-icing/deicing technologies [6], and industrial waste heat recovery [7], owing to their all-solid-state architecture, maintenance-free operation, and high reliability.

Nevertheless, the intrinsic intermittency and fluctuations in solar irradiation severely limit the stable operation of STE systems. Dynamic variations in solar intensity can induce hot-side temperature fluctuations in TEGs, resulting in unstable temperature gradients and power-output decay. More critically, the absence of solar input under nighttime or low-irradiance conditions directly interrupts electricity generation and reduces the overall energy-utilization efficiency of STE systems [8]. Therefore, maintaining stable temperature gradients and achieving continuous energy output under dynamic environmental conditions remain critical scientific challenges for STE technologies. Engineering applications of phase-change materials (PCMs) have attracted growing attention as a promising approach to addressing these issues because latent-heat storage and dynamic thermal regulation can buffer transient thermal inputs [9,10]. As shown in Fig. 1A, through reversible phase transitions, PCMs can temporally decouple heat capture from heat release, effectively buffering external thermal fluctuations and maintaining relatively stable temperature gradients [11–13]. Furthermore, incorporating highly photothermal nanofillers, such as carbon nanotubes (CNTs) and MXenes, into PCM matrices enables the synergistic integration of solar harvesting, thermal storage, and heat-transfer regulation, further improving the overall STE conversion efficiency [11]. With the continuous development of this field, PCM-enabled STE technologies are gradually evolving from single-material optimization toward multiscale coupling and system-level integration. The development of this field can be broadly divided into 3 stages. The early stage (2019 to 2022) mainly focused on improving the structural stability and thermal conductivity of PCMs (Fig. 1B to E) [14–17]. Subsequently, during 2023 to 2024, research emphasis shifted toward mesoscale interfacial engineering and heterogeneous functional coupling to enhance solar absorption and heat-transfer efficiency (Fig. 1F and G) [18,19]. More recently, in 2025 to 2026, the field has further evolved toward system-level integration, characterized by multidimensional structural engineering and multifunctional metal–organic framework (MOF)-derived systems (Fig. 1H to J) [20,21]. Recent studies published in *Research* further illustrate the expansion of photothermal and PCMs toward multifunctional and adaptive thermal management. Bioinspired CNT-decorated metal-foam phase-change composites have combined rapid heat dissipation with latent-heat buffering, supramolecular polyurethane networks have integrated intrinsic photothermal conversion with dynamic mechanical functionality, and thermochromic photothermal coatings have enabled season-adaptive solar heating and antioverheating regulation [22–24]. This evolution reflects the transition of PCM-enabled STE systems from conventional material enhancement toward system-synergistic thermal management. We normalized the output voltages in Fig. 1K to an equivalent irradiation intensity of 1 sun using $V_{\text{norm}}=V_{\text{reported}}/\left(G/G_{0}\right)$, where *G*_0_ = 1,000 W·m^−2^. We introduced this first-order normalization only to facilitate visual comparison among studies conducted under different irradiation intensities. Because TEG voltage also depends on thermal resistance, heat loss, PCM phase-transition behavior, device geometry, and cold-side conditions, the normalized values should not be interpreted as rigorous intrinsic performance metrics or used for direct quantitative ranking across different systems. Based on these developments, this review systematically summarizes recent advances in PCM-enabled STE technologies from the perspectives of material design, structural engineering, and system integration, with emphasis on solar harvesting, heat transfer, thermal storage, and electrical output, as well as the key scientific challenges and future development directions of this rapidly evolving field. Unlike previous reviews that focus mainly on photothermal PCM materials, thermal energy storage, or thermoelectric (TE) conversion individually, this review provides an integrated perspective on PCM-enabled STE conversion across multiple levels, including material modification, structural optimization, PCM–TEG system integration, and emerging applications. Such a multiscale framework aims to provide a more systematic understanding of the development and future opportunities of PCM-enabled STE technologies.

**Fig. 1.**
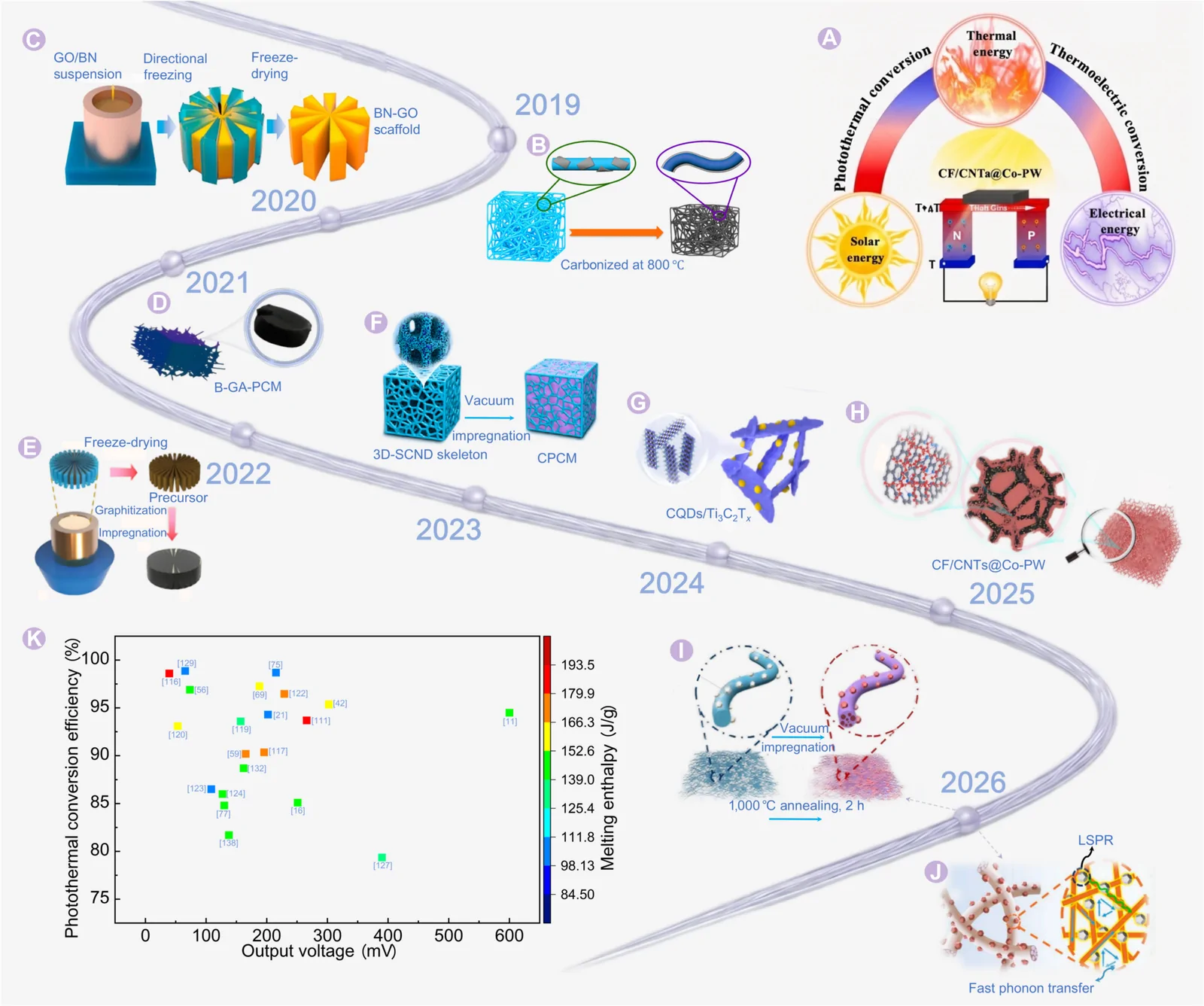
Development road map and performance mapping of phase-change material (PCM)-enabled solar–thermal–electric (STE) conversion technologies. (A) STE conversion mechanism of CF/CNTs@Co-PW [20]. CF, carbon foam; CNTs, carbon nanotubes; PW, paraffin wax. Copyright 2025, Elsevier. (B) Carbonized melamine foam (MF)/reduced graphene oxide (rGO)/BN composite aerogel [14]. Copyright 2019, Royal Society of Chemistry. (C) Graphene oxide (GO)–BN scaffold impregnated with polyethylene glycol (PEG) [16]. Copyright 2020, American Chemical Society. (D) 3-dimensional (3D) interpenetrating-network PCM composite: B-GA-PCM [17]. B-GA-PCM denotes a polyethylene glycol-based phase-change composite supported by black graphene oxide aerogel (B-GA). Copyright 2021, American Chemical Society. (E) Schematic illustration of the fabrication process of structured d-mannitol (DM)/graphene composites [15]. Copyright 2022, American Chemical Society. (F) 3D-SCND framework and corresponding composite PCM [18]. SCND, single-crystal nanodiamond. Copyright 2023, Elsevier. (G) Hybrid hydrogel composed of carbon quantum dots (CQDs)/Ti_3_C_2_T*_x_* [19]. Copyright 2024, Wiley-VCH. Composite PCMs: (H) CF/CNTs@Co-PW [20] and (I) PCNF-ZIF8@PEG [21]. Copyright 2025, Elsevier and 2026, Elsevier. (J) Solar-to-thermal conversion mechanism of PCNF-ZIF8@PEG [21]. PCNF, porous carbon nanofiber. Copyright 2026, Elsevier. (K) Scatterplot showing photothermal conversion efficiency, material enthalpy, and normalized output voltage.

## Fundamental Principles of STE Conversion

### Photothermal conversion mechanisms

As the energy-harvesting component of STE systems, the theoretical efficiency limit of photothermal conversion is fundamentally governed by the response of a material’s electronic (band) structure to electromagnetic radiation [25]. Current photothermal conversion mechanisms can generally be classified into 3 categories: localized surface plasmon resonance (LSPR), interband electronic transitions coupled with nonradiative relaxation, and lattice vibrations accompanied by molecular thermal motion (Fig. 2A) [26]. The fundamental distinction among these mechanisms lies in the manner in which electronic states or band structures interact with incident electromagnetic radiation [25].

**Fig. 2.**
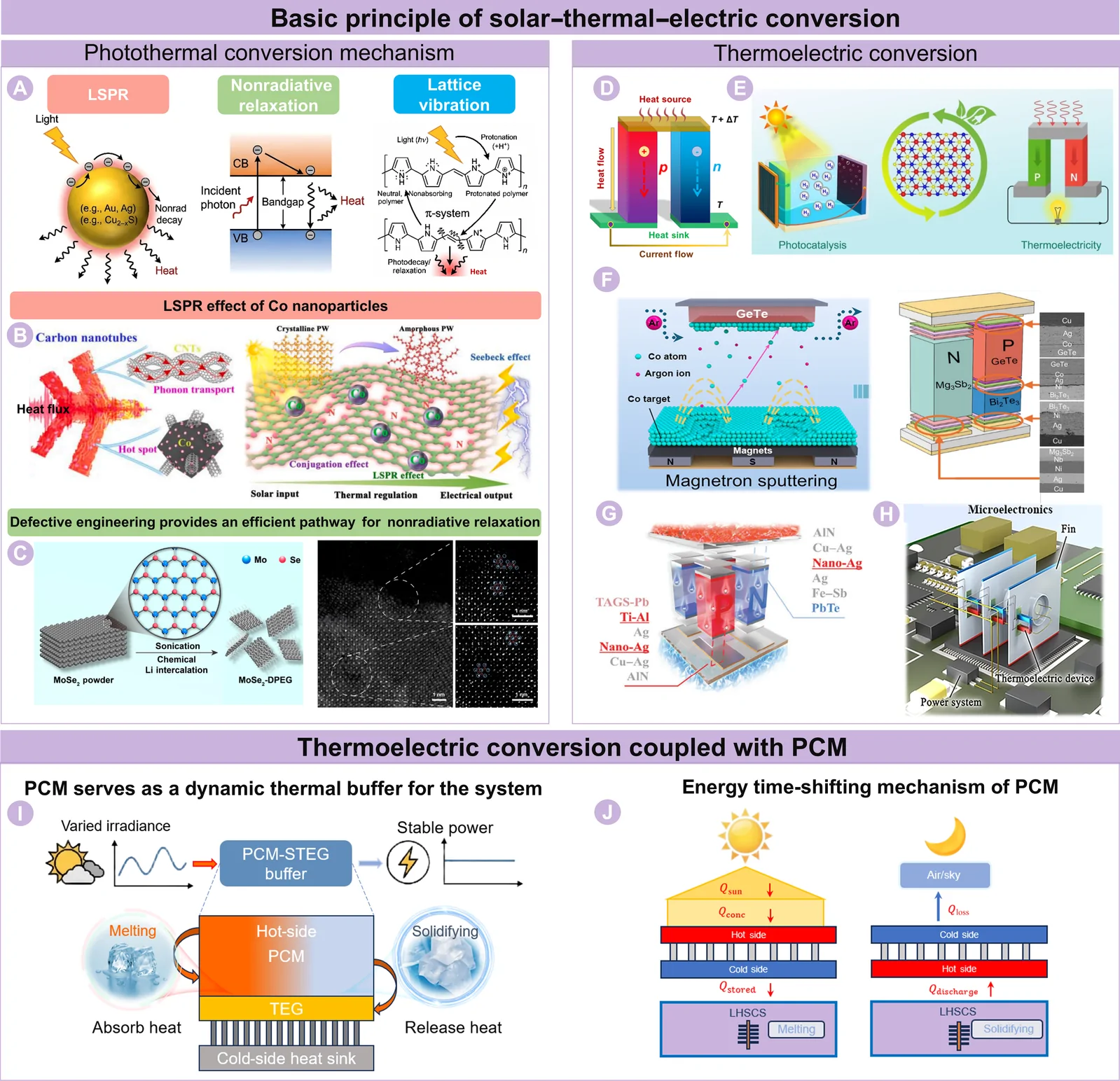
Fundamental principles of solar–thermal–electric (STE) conversion. (A) Schematic illustration of photothermal conversion mechanisms. (B) Co-assisted dual carbon pathways for rapid phonon transport and the corresponding STE conversion mechanism of CF/CNTs@Co-PW [20]. CF, carbon foam; CNTs, carbon nanotubes; PW, paraffin wax. Copyright 2025, Elsevier. (C) High-content metallic 1T phase of MoSe_2_-DPEG and high-angle annular dark-field scanning transmission electron microscopy images of the corresponding edge and in-plane defects [27]. DPEG, distearoyl phosphatidylethanolamine–polyethylene glycol 2000. Copyright 2024, American Chemical Society. (D) Schematic illustration of STE power generation based on the Seebeck effect [110]. Copyright 2020, American Chemical Society. (E) High-efficiency solar-to-hydrogen conversion and complementary thermoelectric (TE) performance of a GeSnS_2_ monolayer [29]. Copyright 2025, American Chemical Society. (F) Schematic illustration of interfacial engineering via magnetron sputtering and GeTe-based TE materials [30]. Copyright 2025, Royal Society of Chemistry. (G) Structural design of TE modules [31]. Copyright 2024, Wiley-VCH. (H) Expanded planar structure for cooling and TE energy conversion [32]. Copyright 2025, National Academy of Sciences. (I) Dynamic thermal-buffering effect and (J) temporal energy-shifting capability of phase-change materials (PCMs) for sustained TE power generation [34]. Copyright 2023, Elsevier.

For plasmonic metals such as Au, Ag, and Cu, as well as heavily doped semiconductors (e.g., Cu_2−*x*_S), incident photons at specific wavelengths induce collective oscillations of free electrons on the material surface, giving rise to the LSPR effect [25]. The subsequent nonradiative decay rapidly converts photon energy into lattice vibrational energy, which is released as heat. Unlike noble metals, Co nanoparticles perform a dual function by enhancing the LSPR effect while simultaneously catalyzing the in situ growth of CNTs and the graphitization of the carbon framework, as evidenced by Raman and x-ray diffraction analyses [20]. Chen et al. [20] demonstrated that the synergistic interaction between catalytically active Co nanoparticles and photophilic carbon foam/CNT carbon hybrid architectures (Fig. 2B) markedly enhances the LSPR effect, facilitating the excitation of photogenerated electrons from low-energy states to higher-energy levels to achieve superior broadband light absorption. Interband electronic transitions and nonradiative relaxation constitute the dominant photothermal conversion pathway in semiconductor materials. When the energy of incident photons exceeds the bandgap energy, electrons are excited from the valence band to the conduction band, generating electron–hole pairs. During carrier recombination, the absorbed energy is dissipated into lattice vibrations through nonradiative relaxation processes. To overcome the limited visible-light absorption of wide-bandgap semiconductors, defect engineering has emerged as an effective strategy. By introducing vacancies and edge defects into the forbidden band, intermediate energy levels can be created, which broaden spectral responsiveness and promote energy dissipation. Huang et al. [27] fabricated defect-rich nanosheets with a high proportion of the metallic 1T phase (an octahedral metallic phase) via a lithium-intercalation-assisted ultrasonic exfoliation strategy (Fig. 2C). The abundant in-plane and edge defects substantially enhanced electron–phonon coupling and markedly improved photothermal performance. Excited electrons subsequently return to their ground states, releasing energy via phonon emission and completing the photothermal conversion process.

Lattice vibrations and molecular thermal motion represent the ultimate pathways for energy dissipation in photothermal systems. In organic macromolecules and eutectic materials, enhanced intramolecular motion can substantially improve the efficiency of nonradiative relaxation. Liu et al. [28] proposed a ternary organic eutectic design strategy in which pyrene was introduced as a third component into a binary organic eutectic system. Acting simultaneously as a molecular spacer and dynamic motion unit, pyrene established active nonradiative transition pathways at the molecular scale. This design substantially intensified molecular thermal motion and lattice friction and enabled highly efficient photothermal conversion in the second near-infrared window.

### TE conversion mechanisms

TE conversion based on the Seebeck effect represents one of the most promising routes for solar TE power generation. As illustrated in Fig. 2D, following photothermal conversion in a solar thermoelectric generation system (STEGS), the temperature difference established between the hot and cold sides drives charge carriers to migrate from the high-temperature region toward the low-temperature region, generating an electrical potential difference and enabling direct energy conversion without moving mechanical components. Solar thermoelectric generators (STEGs) are composed of p-type and n-type TE semiconductor elements. Their open-circuit voltage can be expressed as a function of the temperature difference between the 2 ends and the Seebeck coefficient of the material, as shown in Eq. (1). The open-circuit voltage reflects the Seebeck response under zero-current conditions and should not be directly equated with the deliverable electrical power or conversion efficiency. In practical TEG devices, useful electrical output additionally depends on the internal electrical resistance, load matching, and the time-dependent temperature difference across the device.

$$V_{\text{OC}}=S\times \left(T_{h}-T_{c}\right)$$ (1)

where S is the Seebeck coefficient (V/K), which characterizes the material’s electrical response to a temperature gradient. The conversion efficiency of TE materials is usually determined by the dimensionless TE figure of merit, ZT, which is defined by Eq. (2). Although ZT is a key material-level figure of merit for evaluating TE performance, it does not solely determine device- or system-level conversion efficiency. Device geometry, thermal and contact resistances, electrical loading conditions, and the thermal boundary conditions of the complete system also influence practical performance.

$$\text{ZT}=\frac{S^{2}\sigma T}{\kappa }$$ (2)

where σ is the electrical conductivity, κ is the thermal conductivity, and T is the absolute temperature.

Enhancing TE performance fundamentally relies on multiscale regulation of carrier and phonon-transport processes. Representative approaches include interfacial chemical engineering, band-structure modulation, and evaporation-induced ion migration, all of which aim to achieve efficient cross-scale coupling of TE transport pathways. At the microscopic scale, the anisotropic characteristics and electronic structures of low-dimensional materials can effectively promote charge transport. For example, 2-dimensional (2D) GeSnS_2_ monolayers exhibit an indirect bandgap that enhances in-plane carrier mobility, providing a favorable material platform for multiphysics coupling applications (Fig. 2E) [29]. At the mesoscale, phase stability and internal interface regulation play critical roles in maintaining TE output. Xu et al. [30] developed a GeTe-based TE system using multicomponent doping and magnetron-sputtering engineering. This strategy optimized carrier concentration and electron/phonon-transport pathways while simultaneously suppressing interfacial instability, resulting in improved TE performance (Fig. 2F). At the macroscopic level, reliable interfacial integration and thermal-field management are essential for maximizing energy-conversion efficiency. Cheng et al. [31] introduced nanosilver and Ti–Al diffusion barrier layers into GeTe-based devices. The resulting interfaces suppressed interfacial diffusion, maintained low contact resistance, and substantially enhanced device efficiency (Fig. 2G). Tan et al. [32] proposed a macroscopic strategy based on expanded planar metamaterials, which amplifies local temperature gradients without altering the intrinsic properties of TE materials. This system-level design enhances TE driving forces and shows promise for chip cooling and energy harvesting in microelectronic devices (Fig. 2H). Although substantial progress has been achieved across multiple length scales, current strategies largely remain isolated optimization approaches. A unified design framework capable of simultaneously coordinating carrier, phonon, and ion transport has yet to be established. Consequently, cross-scale coupling efficiency remains limited, and interfacial transport bottlenecks continue to constrain overall system performance.

### PCM-buffered TE conversion

While multiscale transport engineering has been widely deployed to improve the intrinsic performance of TE materials, its real-world efficacy is fundamentally bounded by the ability to establish and maintain stable temperature gradients across the device. Under realistic solar irradiation, transient fluctuations in incident heat flux continuously disrupt sustained temperature differentials, severely eroding the driving force for TE conversion. This system-level limitation cannot be resolved through material-level optimization alone, creating a clear demand for functional thermal management components that can actively buffer thermal input variations. By exploiting latent-heat storage, PCMs act as passive yet robust thermal buffers that reshape thermal-field distributions and stabilize thermal boundary conditions. This buffering function transforms TE conversion from a fluctuation-sensitive process into an output-stabilized energy-conversion regime.

Under dynamic operating conditions, PCMs not only function as static thermal storage media but also operate as dynamic thermal buffers that moderate hot-side temperatures through cyclic latent-heat absorption and release, in turn enabling consistent, steady electrical output under variable solar input (Fig. 2I). Lv et al. [33] used a 3-dimensional (3D) transient model to show that PCM-induced peak shaving suppresses temperature fluctuations and simultaneously enhances PV and TE conversion efficiencies relative to conventional PV–TE systems. Moreover, the temporal energy-shifting capability of PCMs enables electricity generation beyond daylight hours. Montero et al. [34] developed a bidirectional STEG (Fig. 2J) in which the PCM-based latent-heat-storage unit served as a heat sink during the day and a heat source at night. The system enabled both daytime and nighttime electricity generation, although the simulated nighttime contribution under Atacama Desert conditions accounted for only about 0.6% of the annual electricity generation.

### Thermophysical regulation and performance evaluation metrics

The performance of PCM-buffered STE systems should be evaluated through both material-level thermophysical properties and device-level transient responses. Thermal conductivity governs the rate and spatial uniformity of heat transport, whereas latent heat determines the amount of energy that can be buffered during phase transition. Optical absorption and photothermal conversion efficiency describe a composite phase-change material’s (CPCM’s) ability to convert incident radiation into heat, but neither quantity alone determines TE performance. For a TEG, the decisive state variable is the time-dependent temperature difference, which is determined by the coupled thermal resistance and thermal capacitance of the absorber, CPCM, interface, TEG, and cold-side pathway. Consequently, increasing conductivity can accelerate heat delivery to the TEG, but excessive heat spreading or rapid discharge can shorten the duration of a useful temperature gradient. Likewise, high latent heat increases thermal inertia and can suppress short-term temperature spikes, but an inappropriate phase-transition temperature or slow heat-transfer kinetics can delay the release of useful heat. These competing effects indicate that CPCM design should target thermal-impedance and time-scale matching rather than maximizing any single material property. Pure PCMs remain limited by leakage, low thermal conductivity, and, in some systems, supercooling; directional conductive networks and heterogeneous nucleation sites can partially address these limitations [20,35–37]. More generally, the electrical performance of PCM–TEG systems depends on the coupled regulation of interfacial and overall thermal resistance, transient heat transfer, and the resulting time-dependent temperature difference across the TEG. Accordingly, minimizing thermal resistance does not necessarily maximize electrical output, because excessive heat transfer may weaken or shorten the temperature gradient required for sustained TE conversion. Interfacial/contact thermal resistance can be evaluated from the temperature drop across an interface and the corresponding heat flow, i.e., $R_{c}=∆T_{c}/Q$, with thermocouples or infrared thermography used to resolve the interfacial temperature difference. A 3-*I*–*V*-curve method has also been proposed for thermoelectric modules (TEMs), yielding a contact resistivity of 3.57 × 10^−4^ m^2^·K·W^−1^ for a commercial Bi–Te module [38]. In PCM-integrated TE systems, the PV–PCM and PCM–TE contact resistances have been explicitly incorporated into thermal-resistance models, demonstrating that interface regulation can substantially affect heat-transfer behavior and electrical output [39].

The representative studies in Table 1 indicate that high STE performance cannot be attributed to a single material parameter. Increasing thermal conductivity facilitates heat transfer, whereas latent-heat capacity regulates the duration of thermal buffering. Architectural strategies such as continuous conductive networks, asymmetric heat-transfer pathways, and bioinspired interfaces further determine how the absorbed heat is spatially and temporally delivered to the TEG. Importantly, rigorous cross-study normalization remains difficult because device-level quantities such as irradiation intensity, power density, temperature difference, integrated electrical energy, and test duration are sparsely and inconsistently reported, often under different device geometries, electrical loads, and thermal boundary conditions. Accordingly, Table 1 should be interpreted as a summary of reported performance under the original test conditions rather than as a direct quantitative ranking across different studies.

**Table 1.** Main evaluation indices of CPCMs in STE systems

Material system	Thermal conductivity (W/m·K)	Melting enthalpy (J/g)	Photothermal conversion efficiency (%)	Optical absorption efficiency (%)	Spectral absorption range (nm)	Output voltage/current	Ref.
Cu_2−*x*_S/Cu_2_O/*n*-eicosane	ND	68.4	96.10%	ND	200–2,500	ND	[6]
M-SSPCMs	ND	149.5	94.50%	97%	250–2,500	750 mV (1.25 sun)	[11]
*n*-Tetracosane @TiO_2_/Ti_2_O_3_	0.509	144.5	93.70%	ND	200–2,500	149.8 mV	[12]
DM/graphene	8.8	199	95.20%	ND	ND	3,210 mV	[15]
BN/PEG	2.94	147.5	85.10%	ND	ND	251 mV	[16]
3D-SCND/eicosane	1.53	203.5	90.2%	ND	450–800	149.5 mV	[18]
CF/CNTs@Co-PW-3	0.85	146.36	97.07%	ND	200–2,000	246.9 mV	[20]
PCNF-ZIF8@PEG	0.52	99.7	94.3%	ND	200–2,500	202 mV (1 sun)	[21]
TiN-CPCMs	0.88	287.8	90.66%	ND	ND	860 mV	[35]
PPG4	4.36	ND	ND	95%	200–2,500	289 mV (1 sun)	[37]
1,181 mV (5 sun)							
CPAN@CNT/GA	0.764	190.1	93.70%	ND	200–2,500	265.8 mV (1 sun)	[111]
455.6 mV (2 sun)							
541.9 mV (3 sun)							
PEG@NSH	0.888	159	95.36%	93%	250–2,500	907 mV (3 sun)	[42]
TD/ACW/MG-8	0.77	117.6	89.83%	ND	ND	196.6 mV	[76]
Rh@CrO*_x_*/CoO*_y_*STO	ND	ND	ND	ND	250–800	404 mV (5 sun)	[84]
634 mV (10 sun)							
790 mV(13 sun)							
MA@PEG	ND	172.9	90.20%	ND	ND	165.3 mV (1 sun)	[59]
527.2 mV (5 sun)							
CF@CC@PW	4.3	94.5	ND	ND	ND	89.4 mV	[115]
BN	3.24	250.4	ND	ND	250–2,500	ND	[102]
TP-PxBN	3.26	137.2	ND	ND	400–780	271 mV (8 sun)	[72]
D-LA/EG	5.62	193.2	98.60%	ND	ND	315 mV (8 sun)	[116]
PEG@MHP-82	0.235	177.8	90.36%	97%	220–2,500	587 mV (3 sun)	[117]
MF/GO/SAT	0.515	251.5	ND	81.50%	ND	ND	[118]
PEG-BC@MoS_2_/CuS	ND	125.7	93.60%	ND	200–2,000	157.0 mV (1 sun)	[119]
247 mV (3 sun)							
307.0 mV (4 sun)							
T-TME@MCG25	0.73	154.9	93.10%	ND	200–2,500	213.0 mV (4 sun)	[120]
CNiF/PA	ND	68.14	92%	ND	ND	ND	[121]
MoS_2_/Mt-PEG	ND	169.16	96.47%	ND	220–2,500	458 mV (2 sun)	[122]
PW/0.8 g GO	ND	102.1	86.50%	86%	200–800	108.6 mV (1 sun)	[123]
Cu-OH-H/P	2.35	140	96.90%	97%	380–1,600	73.3 mV (1 sun)	[56]
PPy/PEG	0.257	142.4	86.00%	ND	250–2,500	318 mV (2.5 sun)	[124]
MXene@PTA/HDPE/PA6	ND	165	ND	ND	ND	ND	[73]
PW/NiG*_x_*	1.2	164.4	87.90%	ND	360–720	800 mV	[125]
SFPCMs	0.38	125.6	92%	ND	ND	ND	[106]
PEG/rGO(x)PPy(y)	0.75	161.9	97.27%	ND	200–800	187.9 mV (1 sun)	[69]
225.3 mV (1.5 sun)							
PW/OBC/EG	17.12	ND	ND	97.47%	ND	ND	[126]
MF@PPy-PODS/GNP3/PW	0.59	130.61	79.36%	ND	200–800	780 mV (2 sun)	[127]
910 mV (4 sun)							
PW/SEBS/LGA	0.41	217.6	91.80%	ND	ND	ND	[46]
CBNP@LA	0.333	163.2	61%	ND	ND	730 mV (1.5 sun)	[128]
MN-PPG	0.484	110.45	98.70%	ND	ND	215.3 mV (1 sun)	[75]
255.5 mV (2 sun)							
315.4 mV (3 sun)							
SA/PSC800	3.2	105.9	98.87%	ND	220–2,500	196.6 mV (3 sun)	[129]
PEG/polyurethane	ND	145	ND	ND	ND	ND	[130]
EG/OP70-EPDM	1.106	126.8	ND	ND	ND	ND	[131]
PMS-rGO	ND	143	88.70%	95%	ND	810 mV (5 sun)	[132]
1,300 mV (9 sun)							
C−PI/KNF@PEG/CoFe_2_O_4_@PPy	0.283	150	84.80%	ND	200–2,500	259.7 mV (2 sun)	[77]
1-TD/PEG	0.5858	179.79	ND	ND	ND	10.44 mA	[133]
GLBWP	ND	180	ND	ND	ND	ND	[134]
PCC-20	21.3	213.47	87.05%	95%	ND	1,415 mV	[135]
ZnO/CuO/PW	0.31	ND	ND	ND	190–400	ND	[136]
PEDOT:PSS/GO/GNP	1.12	211.5	93.50%	ND	ND	10.16 mV	[52]
PEG/1-TD	0.5828	180.17	ND	ND	ND	12.45 mA	[137]
CNTs@PC(Zn*_x_*Co_1−*x*_)/PEG	ND	ND	98.10%	ND	ND	500 mV	[43]
PU-ssPCM	ND	105.3	ND	ND	ND	ND	[78]
SBR/EO/P	ND	144.98	81.70%	ND	250–2,500	1,100 mV (8 sun)	[138]
GE/PW	0.262	199.3	ND	ND	200–2,500	ND	[139]
SBTP	0.45	145.1	ND	95%	250–2,500	423 mV (3 sun)	[140]
GCSL*_x_*-P	24.02	ND	92.70%	ND	220–2,500	151.3 mV (1 sun)	[54]
330.1 mV (2 sun)							
694.3 mV (3 sun)							
1-TD/GCA	ND	213.57	ND	ND	ND	ND	[141]
PCHSM/PCCSM	ND	112.9	ND	ND	400–500	25.2 mA	[142]
G-Cu/PW	2.99	ND	ND	ND	ND	140 mV (5 sun)	[143]
EG/PW	ND	ND	ND	ND	ND	2,523 mV (1 sun)	[144]
FB-PTEM	0.037	ND	ND	ND	ND	520 mV (1 sun)	[98]
PAG150	8.75	139.8	ND	ND	200–2,500	281.1 mV (1 sun)	[66]
339.7 mV (1.5 sun)							
1,720.1 mV (10 sun)							
2,042.3 mV (12 sun)							
WG/PW	1.7	84.5	95.4%	ND	ND	90.8 mV (1 sun)	[145]

1-TD, 1-tetradecanol; ACW, acid-treated carbonized wood; BC, bacterial cellulose; CBNP, carbon black nanoparticle; CC, coral-like carbon; CF, carbon foam; CNiF/PA, magnetic graphitic carbon/nickel composite foam/paraffin; CNTs, carbon nanotubes; CPAN, cyclized polyacrylonitrile; C–PI, carbonized polyimide; CPCMs, composite phase-change materials; D-LA/EG, double-layered lauric acid/expanded graphite; DM, d-mannitol; EG, expanded graphite; EPDM, ethylene-propylene-diene monomer; EO, oleoyl chloride-modified eumelanin; FB-PTEM, highly flexible and breathable photo-thermal-electric membrane; GA, graphene oxide aerogel; GCA, graphene/cysteamine aerogel; GE, graphene; GLBWP, glycerol–beeswax–paraffin composite; GNP, graphene nanoplatelet; GO, graphene oxide; HDPE, high-density polyethylene; KNF, Kevlar nanofiber; LA, lauric acid; LGA, ultralight flexible graphene aerogel; MA, MXene aerogel; MF, melamine foam; MN-PPG, micro/nanostructured polyethylene/poly(ethylene oxide)/graphene; M-SSPCMs, MXene-integrated solid–solid phase-change materials; Mt, montmorillonite; ND, no data; NSH, secondary hierarchical structure formed in nickel foam using a silica-fiber scaffold and polypyrrole-modified halloysite nanotubes; OBC, olefin block copolymer; PA6, polyamide 6; PC, porous carbon; PCCSM, phase-change cool-storage material; PCHSM, phase-change heat-storage material; PCNF, porous carbon nanofiber; PEDOT:PSS; poly(3,4-ethylenedioxythiophene) polystyrene sulfonate; PEG, polyethylene glycol; PMS, paraffin-loaded melamine sponge; PODS, polymerized octadecylsiloxane; PPG4, paraffin-impregnated PG4 graphene aerogel composite; PPy, polypyrrole; PU-ssPCM, self-supporting polyurethane-based solid–solid phase-change material; PW, paraffin wax; rGO, reduced graphene oxide; SA, stearic acid; SAT, sodium acetate trihydrate; SBR, styrene-butadiene rubber; SBTP, silylated bacterial cellulose/hydroxylated carbon nanotube/polyethylene glycol; SCND, single-crystal nanodiamond; SEBS, styrene-*b*-(ethylene-*co*-butylene)-*b*-styrene; SFPCMs, superflexible phase-change materials; STE, solar–thermal–electric; TD, tetradecanol; TP-PxBN, double-layered composite phase-change material obtained by impregnating polyethylene glycol into a TP-SxBN scaffold, where TP stands for thermochromic particles and x represents the mass of boron nitride (BN) in the slurry; T-TME@MCG25, surface-treated TME@MCG25 composite (TME = 1,1,1-tri(hydroxymethyl)ethane), obtained through photothermal etching followed by hydrophobic polydimethylsiloxane coating; WG, worm graphite

To provide a clearer perspective on the development of this field, Fig. 3 summarizes the research framework and evolutionary trajectory of PCM-enabled STE technologies, from materials engineering to the expansion of applications.

**Fig. 3.**
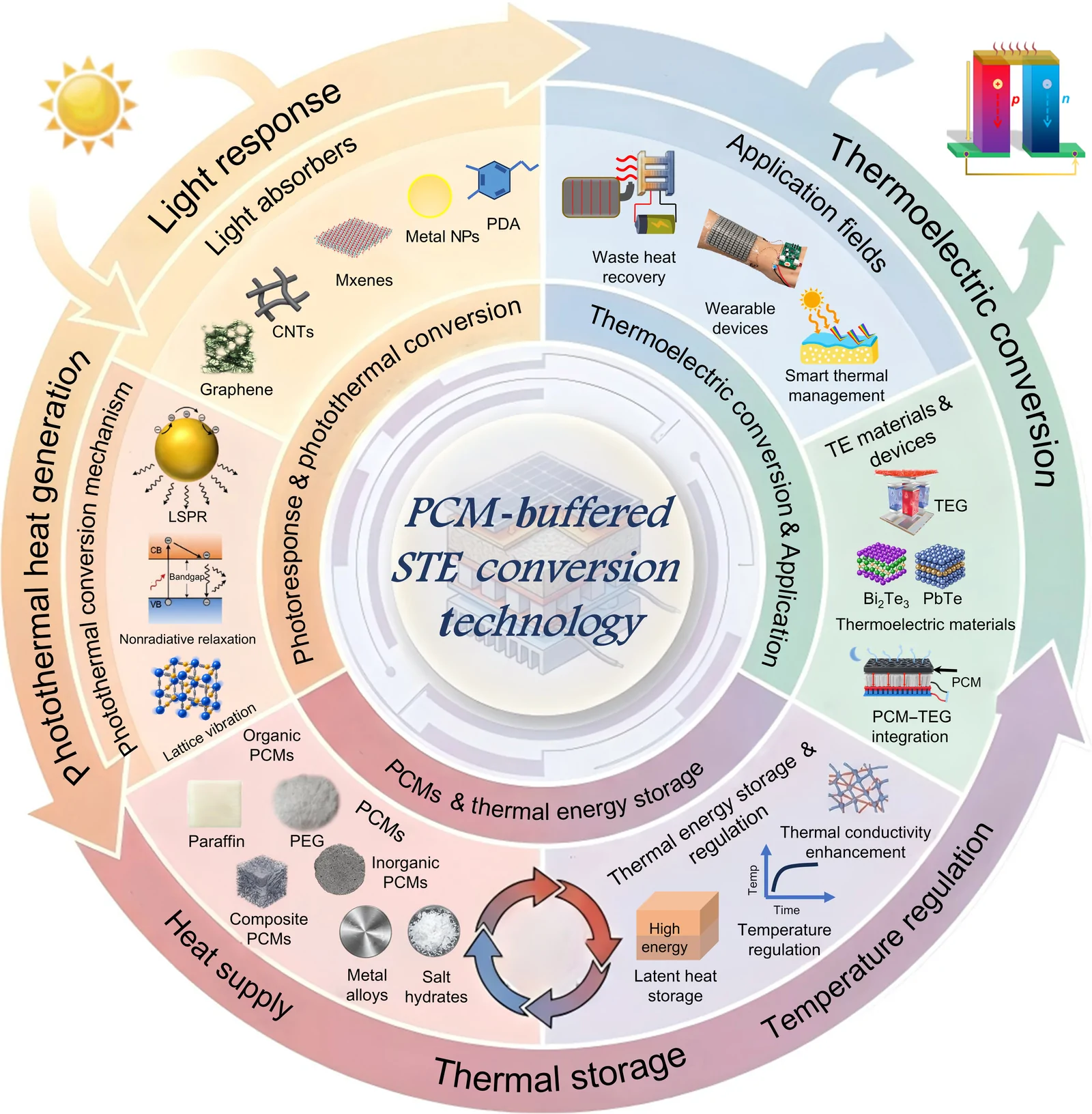
Solar–thermal–electric (STE) conversion technology from material design to application scenarios, including photothermal heat generation, photothermal conversion mechanisms, phase-change materials and thermal energy storage, temperature regulation, thermoelectric conversion, and practical applications [4,24,31,102,110,111]. Copyright 2026, Elsevier; 2026, AAAS; 2024, Wiley-VCH; 2020, American Chemical Society; 2025, Elsevier; and 2024, Wiley-VCH.

## Material-Level Modification: Construction of Photothermal CPCM Systems

Rapid thermal response is a fundamental prerequisite for stable, consistent TE output in STE energy-storage systems. Conventional organic PCMs, however, are technically constrained by 2 inherent limitations: weak intrinsic solar-light-harvesting capability and low thermal conductivity. Together, the 2 limitations prevent direct use as standalone photothermal conversion media. To address these bottlenecks, a variety of composite engineering strategies have been proposed, including layered assembly [40], microencapsulation [12], scaffold confinement [41], compositional doping [42–44], and surface coating [45]. Broadly, composite-modified organic PCMs fall into 4 major material families: carbon-based composites, polymer-based composites, inorganic/metal-based composites, and micro/nano-confined composite systems (Fig. 4A).

**Fig. 4.**
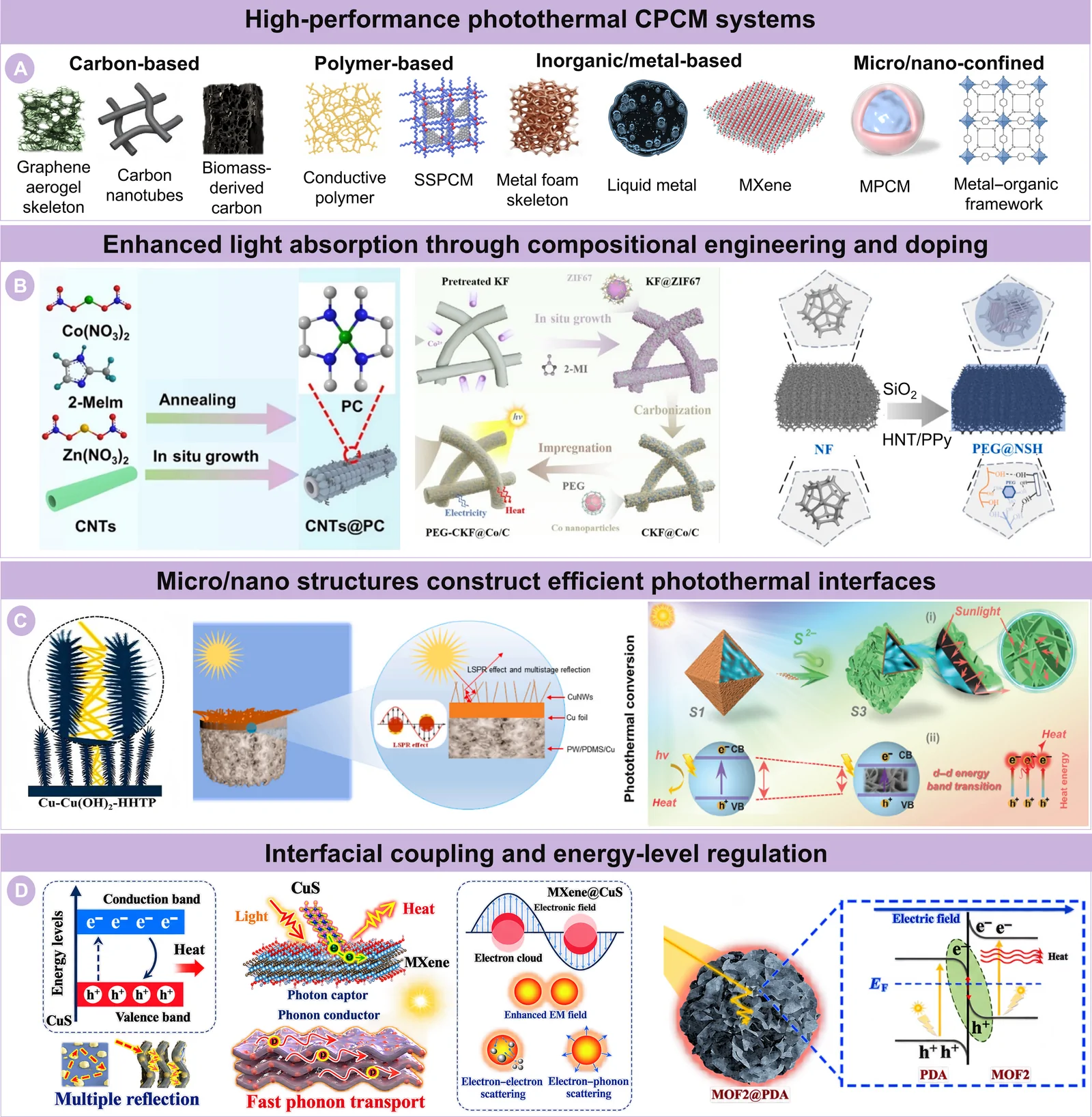
High-performance photothermal composite phase-change material (CPCM) systems. (A) Representative material platforms, including carbon-based CPCMs [111], polymer-based CPCMs [78], inorganic/metal-based CPCMs, and micro/nano-confined CPCMs [12]. Copyright 2025, Elsevier; 2025, Wiley-VCH; and 2025, Wiley-VCH. (B) Enhanced light absorption through compositional engineering and doping, including Co-decorated CNTs@PC [43], hollow porous carbon-fiber tubular structures uniformly decorated with Co nanoparticles [44], and polypyrrole (PPy)-modified PEG@NSH composites [42]. CNTs, carbon nanotubes; NSH, secondary hierarchical structure formed in nickel foam using a silica-fiber scaffold and polypyrrole-modified halloysite nanotubes; PC, porous carbon; PEG, polyethylene glycol. Copyright 2023, John Wiley & Sons; 2025, Wiley-VCH; and 2025, Elsevier. (C) Micro/nanostructural engineering for efficient photothermal conversion, including multiple reflection and scattering induced by Cu-OH-H hierarchical structures [56], photothermal conversion in CF@CuNWs systems [74], and photothermal enhancement in microencapsulated phase-change materials (MPCMs) [6]. CF, carbon foam; CuNWs, copper nanowires. Copyright 2025, Elsevier; 2026, Elsevier; and 2023, Wiley-VCH. (D) Interfacial coupling and energy-level regulation for enhanced photothermal conversion, including the photothermal conversion mechanism of PEG-MXene@CuS through interfacial coupling, nonradiative relaxation, and synergistic localized surface plasmon resonance (LSPR) effects [63], and the heterointerface-mediated charge transfer and energy-level regulation in Co-MOF@PDA [64]. MOF, metal–organic framework; PDA, polydopamine. Copyright 2026, Springer Nature, and 2026, Elsevier.

### Carbon-based CPCMs

Carbon-based phases are attractive because broadband optical absorption and high intrinsic thermal conductivity can be combined in a lightweight filler platform. At the material level, the key variables are carbon dimensionality, defect density, graphitization degree, surface chemistry, and filler–filler/filler–PCM contact quality. Graphene-based fillers can lower intrinsic conduction resistance, whereas CNTs can bridge neighboring conductive domains and reduce transport-path discontinuities. However, increasing carbon loading also replaces active PCM volume and may reduce latent-heat density. Carbon modification should therefore be evaluated through the gain in heat-transfer rate relative to the loss in storage capacity, rather than through thermal conductivity alone [37,46].

This trade-off is evident in recent carbon–metal and biomass-derived systems. Co-functionalized carbon networks can combine broadband photothermal absorption with improved interfacial heat transfer and generate a stable output voltage of 246.9 mV under 1-sun irradiation [20]. Biomass-derived carbons provide low-cost, chemically tunable surfaces and naturally continuous channels; reported precursors include agricultural residues, wood- or fiber-derived structures, and fungal scaffolds [47,48]. Rather than treating these precursors as separate material categories, their common advantage lies in preserving a carbonized framework with abundant light-trapping interfaces and continuous heat-transfer pathways. Surface functionalization can further tune PCM affinity and interfacial resistance [44,49]. Thus, the transferable design rule is to construct a chemically compatible, low-resistance conductive phase while retaining sufficient PCM fraction for latent-heat storage.

### Polymer-based CPCMs

Conventional solid–liquid PCMs are susceptible to leakage during phase transitions [50]. Organic polymer systems provide an effective solution through molecular structure design and spatial confinement, enabling intrinsic improvements in structural stability and interfacial compatibility [51]. Conjugated conductive polymers, including poly(3,4-ethylenedioxythiophene) polystyrene sulfonate (PEDOT:PSS) and polypyrrole (PPy), exhibit strong near-infrared absorption owing to dopant-induced charge carriers. As highly efficient photothermal conversion units, these materials can be integrated into porous scaffolds to simultaneously confine PCM cores and establish continuous heat-transfer pathways. Consequently, PEDOT:PSS/graphene oxide/graphene nanoplatelet/polyethylene glycol (PEG) composites reached a thermal conductivity of 1.12 W·m^−1^·K^−1^, a 262.7% increase over that of pure PEG [52].

Solid–solid phase-change materials (SSPCMs) provide a complementary route because phase transition proceeds without a macroscopic liquid phase. Luo et al. [35] incorporated TiN nanocrystals into a pentaerythritol-based SSPCM; the optimized composite exhibited a latent heat of 287.8 J/g, retained 96.06% of the energy-storage density of pure pentaerythritol, and achieved a photothermal conversion efficiency of 90.66%. The distinction between energy-storage retention and photothermal conversion efficiency is important when comparing material metrics. Conductive additives can also introduce electrothermal functionality, but the accompanying displacement of PCM reduces storage density [53]. These results illustrate a general material-level trade-off: optical/electrical functionality should be introduced without allowing the functional phase to dominate the thermal-storage volume.

### Inorganic/metal-based CPCMs

Heat-conduction mechanisms differ across material classes: electronic transport contributes substantially in metals, whereas phonon transport dominates in many nonmetallic inorganic fillers and carbon-based frameworks. In parallel, the associated LSPR effects markedly enhance light absorption, promoting rapid thermal response and stable electrical output in STE systems. Highly thermally conductive metals, such as copper and nickel, can accelerate the thermal response of PCMs and support rapid heat transfer and efficient thermal storage under transient solar irradiation [42]. Liquid metals provide additional advantages through a unique combination of fluidity and metallic-level thermal conductivity, enhancing thermal transport while preserving the latent-heat utilization capability of PCMs [54]. For high-temperature thermal-energy-storage applications, alloy-based materials, such as Ni–Mn–Ti systems, exhibit large latent heat and excellent thermal cycling stability, making them promising candidates for high-temperature PCM systems [55]. Notably, Huang et al. [56] fabricated a pine-needle-inspired hierarchical porous scaffold through a self-assembly strategy. The resulting architecture not only strengthened interfacial adhesion but also enhanced light harvesting through multiple scattering, ultimately achieving a photothermal conversion efficiency of up to 96.9%.

Among emerging inorganic materials, MXenes and other 2D transition-metal carbides/nitrides have evolved from conventional conductive fillers into key components for interfacial engineering [11,57,58]. Metallic conductivity, abundant surface functional groups, and excellent photothermal responsiveness enable MXenes to simultaneously enhance thermal transport by constructing 3D heat-conduction networks [11,59] and improve compatibility with PCM matrices through surface chemical interactions. The combined interfacial and transport effects can expand both latent-heat utilization efficiency and the operational temperature range [58]. The resulting performance gain arises from the combination of intrinsic conductivity and reduced local interfacial resistance, emphasizing that interface quality must be considered alongside filler conductivity. Accordingly, interface engineering should be evaluated by whether it reduces the dominant local resistance and increases the useful transient heat flux to the TEG, rather than by the additive’s conductivity alone.

### Micro/nano-confined CPCMs

For precise encapsulation and functional customization of PCM cores at micro- and nanoscales, microencapsulation and MOF-based technologies have emerged as highly promising approaches. By constructing physical barriers or nanoscale confinement channels, these strategies effectively suppress leakage while introducing a variety of intelligent functionalities. Microencapsulation technology utilizes core–shell architectures to achieve both PCM confinement and functional integration. In addition to preventing leakage, shell engineering enables the incorporation of enhanced photothermal conversion, flame retardancy, and environmental responsiveness [60]. Representative inorganic shell materials include TiO_2_, SiO_2_, and copper oxides, whereas organic shell materials include poly(methyl methacrylate), melamine-formaldehyde resin, and polyurethane. As a result, the design philosophy of microencapsulated phase-change materials (MPCMs) has evolved from simple thermal storage units toward multifunctional intelligent systems incorporating self-healing, flame-retardant, and anti-icing and deicing capabilities. Hou et al. [6] developed dual-shell MPCMs that function not only as anti-icing barriers but also as highly efficient photothermal conversion platforms. By inducing multiple photon reflections and absorptions within the internal cavity, the composite achieved a photothermal conversion efficiency of 96.1% and a thermal conductivity of 7.523 W·m^−1^·K^−1^, corresponding to an 812.8% increase relative to pure *n*-eicosane.

Compared with conventional MPCM encapsulation, MOF-based systems offer more sophisticated confinement through highly tunable nanoscale topological pore structures. Benefiting from pore-confinement effects and strong van der Waals interactions, MOFs provide enhanced shape stability and PCM retention without appreciably compromising latent heat. PCNF-ZIF8@PEG exhibited melting/freezing latent heats of 99.7/96.4 J·g^−1^ and maintained reliable phase-change performance over 100 heating/cooling cycles, with a capacity-fading rate of only approximately 0.035% per cycle. The composite also achieved a solar–thermal conversion efficiency of 94.3% and generated an electrical output of 202 mV under 100 mW·cm^−2^ simulated solar irradiation [21]. However, pristine MOF frameworks generally suffer from weak solar absorption and low thermal conductivity. To address both limitations, surface functionalization and high-temperature derivatization have become the 2 dominant modification strategies. Surface functionalization using polydopamine [61] or PPy [62] can substantially enhance optical absorption. Alternatively, high-temperature derivatization transforms MOFs into thermally conductive carbon frameworks and simultaneously generates metallic nanoparticles in situ, thereby enabling the synergistic enhancement of coupled STE performance.

Overall, high-performance photothermal CPCM systems are evolving toward multifunctional integration and precise multiscale structural regulation. Performance enhancement arises primarily from 3 fundamental mechanisms. First, compositional engineering and doping strategies regulate the electronic structure and photon–matter interactions, thereby broadening spectral absorption and enhancing photothermal conversion efficiency (Fig. 4B). Second, micro/nanostructural engineering establishes continuous thermal conduction networks and highly efficient interfaces, optimizing both light-harvesting and heat-transfer pathways (Fig. 4C). Third, interfacial coupling and energy-level regulation modify interfacial electronic states and promote charge/excitation transfer followed by nonradiative relaxation, providing an additional route for broadband photothermal conversion (Fig. 4D) [63,64]. These mechanisms can coexist and synergistically govern photon absorption, energy dissipation, and heat transport in advanced CPCMs. The synergistic interplay among these mechanisms has driven the evolution of CPCMs from passive thermal-storage materials to integrated STE conversion platforms. Although carbon-based materials continue to dominate owing to a balanced overall performance profile, emerging inorganic and metallic materials offer new opportunities to overcome the intrinsic limitations of single-component systems. Meanwhile, microencapsulation technologies and MOF-derived strategies enable precise confinement of PCM cores at the micro- and nanoscales, offering innovative pathways to overcome current performance bottlenecks and advance next-generation CPCMs.

## Structural-Level Optimization: Multiscale Architectural Design and Functional Synergy

Conventional CPCMs often struggle to simultaneously satisfy the requirements of high solar absorption, high thermal conductivity, and excellent shape stability. Structural engineering has therefore emerged as a critical strategy for balancing the competing properties and enhancing overall system performance [65]. Inspired by naturally occurring architectures and enabled by advanced manufacturing technologies, researchers have developed a variety of structural designs, including porous aerogels, hierarchical architectures, bioinspired micro- and nanostructures, and multi-stimuli-responsive systems. Collectively, such architectures provide multiscale regulation of photon, phonon, and electron transport, enabling the decoupling and optimization of energy-transfer pathways.

### Porous aerogel architectures

As illustrated in Fig. 5A, aerogels possess ultrahigh porosity, ultralow density, and 3D interconnected pore networks. Graphene-nanoplatelet-based aerogels (Fig. 5B) [66] and cellulose-nanofiber-based aerogels (Fig. 5C) [67] are widely regarded as ideal scaffolds for PCM incorporation and have demonstrated considerable potential for solar-energy applications [68]. The directional construction of ordered frameworks can effectively suppress undesirable heat diffusion while enhancing heat transport along preferred directions. For example, aligned graphene frameworks establish efficient phonon-transport channels along the direction of irradiation and increase the thermal conductivity of CPCMs to 4.36 W·m^−1^·K^−1^ [37]. Furthermore, incorporating conductive polymers such as PPy simultaneously enhances solar absorption and interfacial heat transfer and supports stable TE output voltages of up to 187.9 mV [69]. To achieve precise control over aerogel microstructures, 3D-printing technologies have increasingly been employed to fabricate PCM composite scaffolds with complex geometries (Fig. 5D) [70,71]. By enabling digital design of heat-transfer pathways, 3D printing overcomes the nonuniform stress distribution commonly encountered in conventional impregnation methods, which improves both light-harvesting capability and thermal-transport continuity. However, a denser conductive framework may accelerate heat leakage away from the hot side or reduce the latent storage volume. Porous architecture should therefore be optimized for directional conductance and thermal response time rather than for maximum isotropic conductivity.

**Fig. 5.**
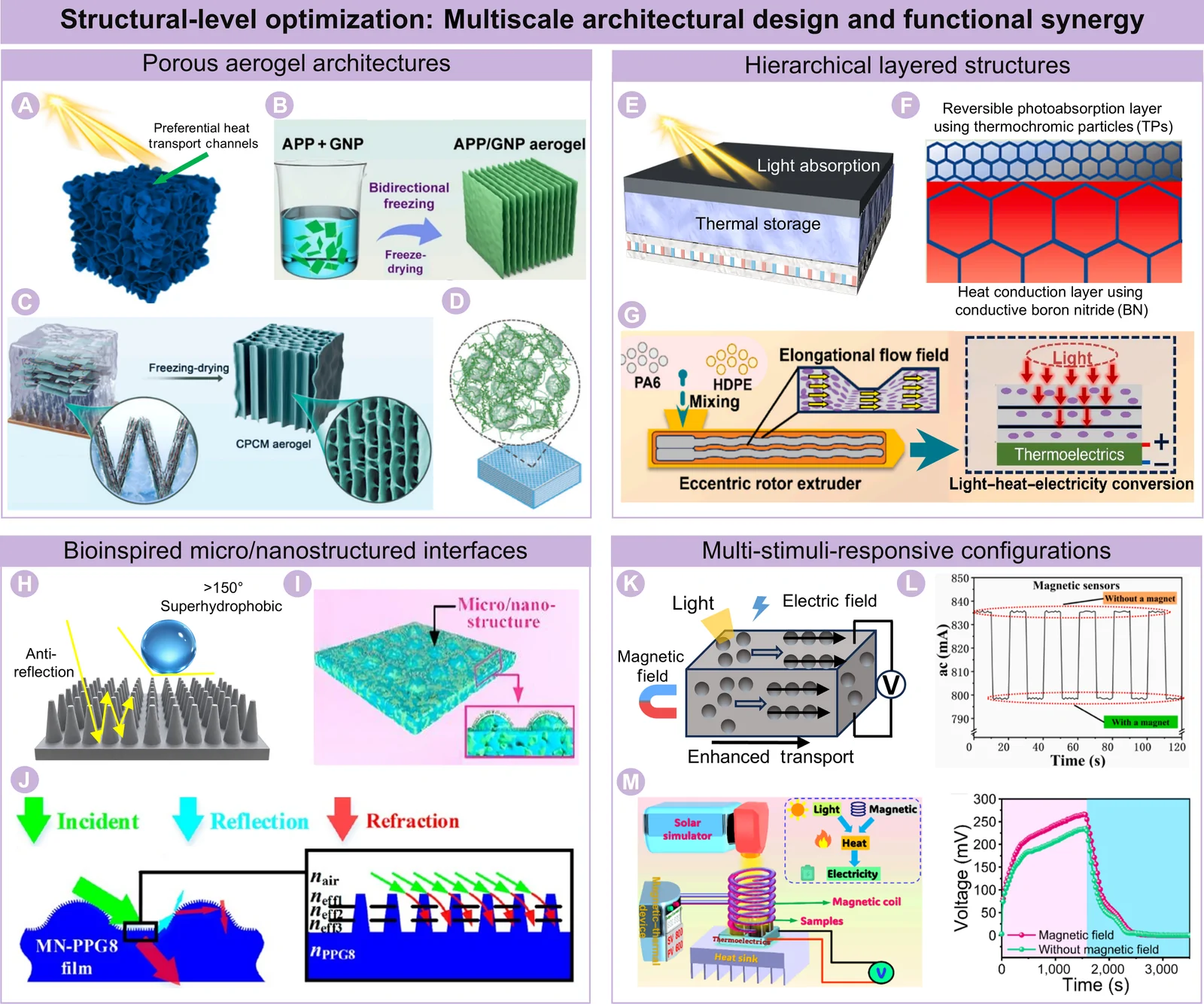
Structural engineering strategies for enhancing solar–thermal–electric (STE) conversion performance. (A) Representative porous aerogel architectures for photothermal composite phase-change materials (CPCMs) [59]. Copyright 2025, Elsevier. (B) Schematic illustration of the fabrication process of ammonium polyphosphate (APP)/graphene nanoplatelet (GNP) aerogels [66]. Copyright 2026, Elsevier. (C) Preparation procedure of CPCM aerogels [67]. Copyright 2025, Elsevier. (D) 3D-printed phase-change material (PCM)/cellulose nanofibril (CNF) composite monoliths [70]. Copyright 2022, Elsevier. (E) Representative hierarchical layered structures for multifunctional energy management. (F) Bilayer composite PCM consisting of a reversible light-absorbing top layer containing thermochromic particles and a thermally conductive bottom layer containing BN [72]. Copyright 2022, Elsevier. (G) Sandwich-structured solid–solid phase-change material (SSPCM) system [73]. Copyright 2025, Elsevier. (H) Representative bioinspired micro/nanostructured interfaces. (I) Bioinspired MN-PPG8 film and (J) its corresponding antireflection mechanism [75]. Copyright 2022, American Chemical Society. (K) Multi-stimuli-responsive configurations for adaptive thermal management. (L) Magnetic responsiveness of tetradecanol (TD)/acid-treated carbonized wood (ACW)/MG-8 composites [76]. Copyright 2023, Elsevier. (M) Schematic illustration of magnetically accelerated STE conversion and the corresponding output-voltage comparison [77]. Copyright 2024, American Chemical Society.

### Hierarchical layered structures

In conventional monolithic composites, photothermal conversion and thermal storage often compete for the same spatial domain, resulting in functional conflicts. Hierarchical architectures, as illustrated in Fig. 5E, address this issue by spatially separating functions. Specifically, the top layer is designed for efficient solar absorption and rapid heat transfer, whereas the underlying thermal-storage layer serves as a heat reservoir and transport medium, collectively enhancing overall energy-utilization efficiency. For example, Tang et al. [72] developed a bilayer architecture featuring a highly conductive thermal-storage layer beneath a reversible light-absorbing layer (Fig. 5F). The bilayer design improved output stability while maintaining sustained power generation. Beyond macroscopic layering, secondary hierarchical structures provide additional opportunities for refined thermal management. Li et al. [42] introduced silica fibers into a nickel foam framework as a secondary supporting layer and subsequently deposited PPy-modified halloysite nanotubes onto the fiber surfaces. The resulting “skeleton-within-a-skeleton” micro-/nanostructured network achieved an output voltage of 907 mV under intense solar irradiation, demonstrating substantially enhanced STE conversion performance. Similarly, Ai et al. [73] proposed a sandwich-structured system in which functional layers were embedded within a polymer matrix (Fig. 5G). The sandwich configuration simultaneously ensured mechanical robustness and enabled synergistic optimization of solar absorption, thermal conduction, and thermal storage. The resulting SSPCM exhibited a latent heat above 165 J·g^−1^ at a phase-change temperature of approximately 130 °C and reached 145.4 °C during photothermal heating. Compared with the reference configuration, the no-light power-generation duration was extended by 16.4%, while the power-generation capacity increased by 47.6%. Importantly, the composite maintained stable thermal performance after 200 thermal cycles. At the architectural level, pathway geometry and latent-heat storage jointly determine the effective thermal impedance and response time, thereby controlling both the magnitude and duration of the time-dependent temperature difference.

### Bioinspired micro/nanostructured interfaces

Inspired by the surface morphologies found in nature, bioinspired micro- and nanostructures have been widely explored for the surface engineering of photothermal PCMs (Fig. 5H). By constructing subwavelength topological features, such structures markedly suppress optical reflection losses, enhance light absorption, and enable simultaneous optimization of optical and interfacial properties. In addition to improved photothermal performance, bioinspired architectures can impart multiple functionalities, including superhydrophobicity, antireflective behavior, self-cleaning capability, and anti-icing and deicing performance [74]. Wu et al. [75] developed a composite film incorporating bioinspired microlens arrays derived from insect compound-eye structures (Fig. 5I). The subwavelength architecture induced multiple refractions and total internal reflections of incident light, effectively minimizing surface reflection and achieving exceptionally low reflectance. Moreover, the film exhibited a water contact angle of 156° and a sliding angle of only 6°, demonstrating excellent superhydrophobicity. Under outdoor operating conditions, the material maintained stable photothermal performance and generated an open-circuit voltage of up to 315.4 mV (Fig. 5J). Importantly, optical enhancement contributes to electrical output only when the added absorbed heat is transported to the TEG without being dissipated laterally; surface topology and subsurface thermal pathways must therefore be co-designed.

### Multi-stimuli-responsive configurations

To overcome the limitations of purely thermally driven systems, multi-stimuli-responsive architectures have been developed by incorporating magnetic, electrical, and photoresponsive components into PCM systems (Fig. 5K). Unlike conventional static structures, the resulting architectures can dynamically respond to external stimuli, undergo adaptive reconfiguration, and substantially expand the application scope of PCMs in intelligent photothermal systems. For magnetic-field-responsive systems, Fang et al. [76] incorporated Fe_3_O_4_ magnetic nanoparticles into a 3D carbon-based photothermal collector, enabling controllable magnetic responsiveness while preserving high photothermal conversion efficiency (Fig. 5L). Building upon this concept, Shi et al. [77] employed a magnetic-field-induced alignment strategy to optimize heat-transfer pathways. Compared with control samples fabricated without magnetic-field assistance, the resulting STEGS exhibited an increase in peak output voltage from approximately 232 to 268 mV, corresponding to an enhancement of about 15.5% (Fig. 5M). The voltage increase demonstrates that magnetic-field regulation can effectively accelerate thermal transport processes and improve TE output. In addition, ultraflexible polyurethane-based PCMs incorporating dynamic cross-linked networks have been developed for adaptive thermal management [78]. The polyurethane-based PCMs exhibit high latent heat and reversible crystalline–amorphous transitions in response to temperature stimuli. Upon photothermal activation, programmable shape morphing can be achieved, providing a new design paradigm for intelligent actuation in flexible devices. Future responsive systems should be evaluated by the magnitude, reversibility, response time, and energy cost of the induced change in thermal impedance.

Across these architectural strategies, the decisive outcome is therefore the time-dependent heat-flow path they create: altered thermal impedance reshapes transient heat flux, which propagates to time-dependent temperature difference and ultimately to voltage, power, and integrated electrical energy. Structural engineering has driven the evolution of solar–thermal PCMs from passive thermal-storage media toward multifunctional, intelligent composite systems that integrate light harvesting, heat transport, leakage suppression, self-cleaning capability, and multi-stimuli responsiveness. Specifically, porous aerogel architectures address the long-standing trade-off between shape stability and lightweight design by rationally regulating pore structure. Hierarchical architectures enable spatial decoupling and synergistic optimization of multiple functionalities, which improves the efficiency of energy capture, storage, and utilization. Meanwhile, bioinspired micro- and nanointerfaces and multi-stimuli-responsive structures substantially enhance environmental adaptability and operational robustness under complex working conditions. By simultaneously regulating photon, phonon, and heat-flow transport pathways across multiple length scales, the combined structural strategies provide a fundamental framework for achieving efficient energy conversion and pave the way toward the advanced system integration strategies discussed in the following.

## System-Level Integration: Coupling Strategies and Performance Optimization of PCM–TEG Integrated Systems

Building on the material-level modification and structural-level optimization detailed in preceding sections, the efficient coupling and integration of PCMs with TEGs has emerged as a critical research frontier in solar thermal energy harvesting [74,79]. The overall performance of STE systems is governed not only by the intrinsic properties of the constituent materials but also by thermal flux management, transient thermal regulation, and diurnal energy scheduling. The following discussion systematically reviews integration strategies and optimization pathways for PCM–TEG integrated systems across 3 dimensions: system architectural design, all-day power generation, and transient thermal-load regulation.

### System architecture design

The primary objective of system architecture optimization is to achieve efficient heat-flow management and transport through precise regulation of spatial configuration, encapsulation geometry, and interfacial coupling [80]. At the macroscopic level, Zhao et al. [81] established a 3D numerical model of a PCM–TEG system (Fig. 6A) to investigate the influence of the relative positions of the heat source and TEM on PCM melting behavior and electrical output. Although incorporating PCMs slightly reduced peak power-generation efficiency, it effectively buffered thermal fluctuations and markedly prolonged stable operating durations. Beyond spatial arrangement, encapsulation geometry also plays a crucial role in maximizing solar-energy utilization. Akshayveer et al. [82] proposed a PCM enclosure featuring expanded and recessed top structures (Fig. 6B). The optimized enclosure increased energy-storage density by about 10%; moreover, the solar-radiation gain, defined in the original study as the ratio of utilized to unutilized radiation, was approximately 17 times that of the conventional PV system, while the PV electrical conversion efficiency approached 12%. At the interfacial level, Armenta-Déu [83] proposed an unconventional biphasic TE architecture that departed from traditional planar TEG configurations (Fig. 6C). Compared with conventional ceramic-encapsulated Peltier cells, the biphasic configuration produced a power-generation ratio of 1.65 to 7.93 depending on the temperature difference and transient time, with a maximum prototype power of 36.5 W versus 7.3 W for the conventional configuration. However, reducing contact resistance or accelerating heat transfer is not inherently beneficial if it simultaneously collapses the hot–cold-side temperature difference; future architectures should therefore co-optimize interface resistance, PCM placement, and cold-side dissipation against time-dependent temperature difference and integrated electrical energy rather than peak heat-transfer rate alone.

**Fig. 6.**
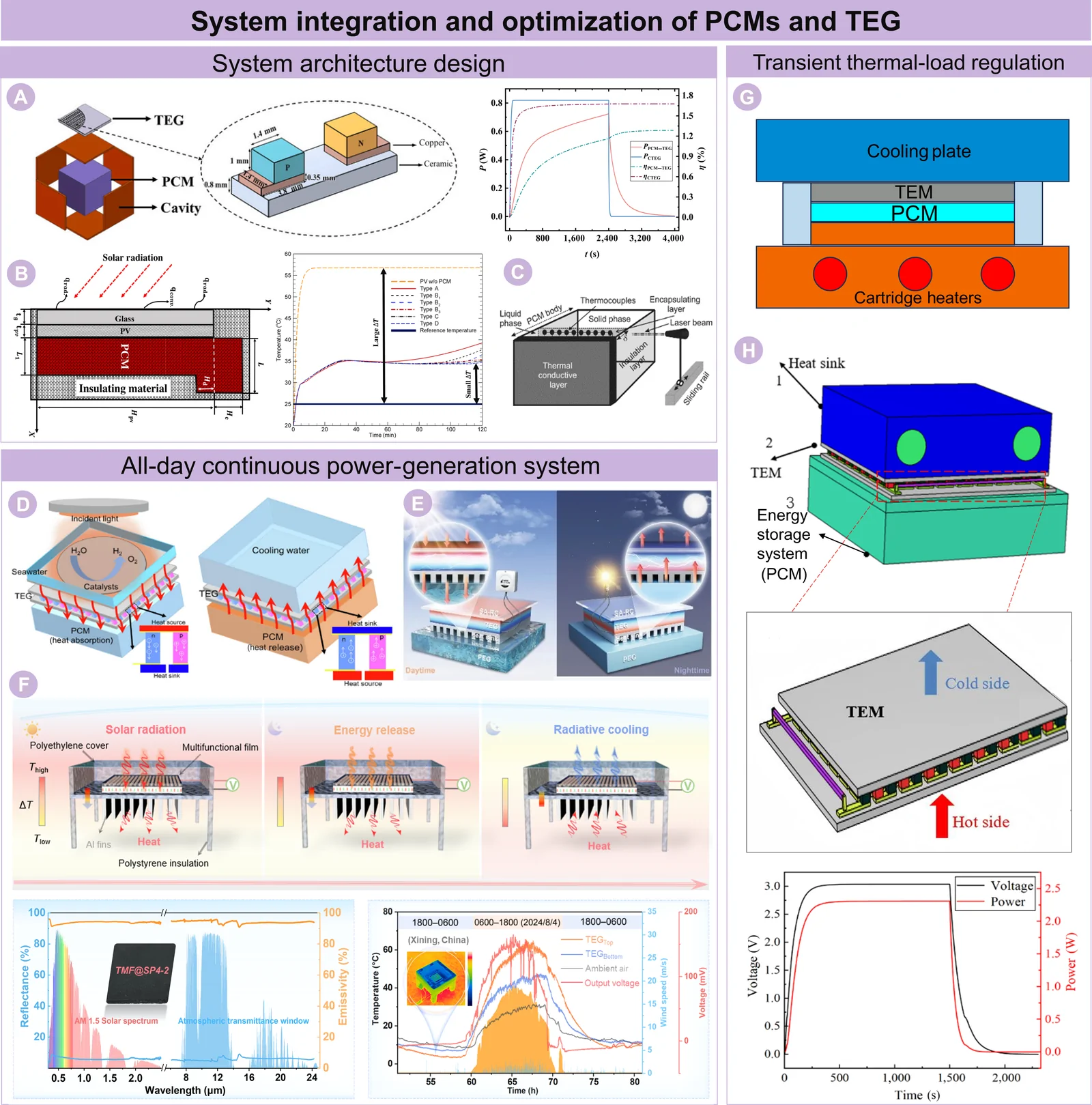
System integration and optimization strategies for phase-change material (PCM)–thermoelectric generator (TEG) systems. (A) Schematic illustration of a PCM–TEG model and comparison of thermoelectric (TE) performance between PCM-assisted TEG and conventional TEG systems [81]. Copyright 2025, Elsevier. (B) 2-dimensional (2D) view of a photovoltaic (PV) panel integrated with a PCM enclosure and the transient variation of average PV-cell temperature in the PV/PCM system [82]. Copyright 2022, Elsevier. (C) Schematic illustration of a Peltier-cell prototype [83]. Copyright 2025, Springer Nature. (D) Schematic diagram of a photothermal catalytic (PTC)–TEG–PCM device operating under daytime illumination and nighttime dark modes, together with the corresponding output voltages under different solar intensities [84]. Copyright 2024, Elsevier. (E) Conceptual illustration of all-day TE power generation based on the synergistic integration of photothermal conversion and radiative-cooling processes [89]. Copyright 2024, Wiley-VCH. (F) Working principle of a TE system, spectral performance of the multifunctional film from the ultraviolet to mid-infrared region, and field measurements of continuous TE operation in Xining, China [12]. Copyright 2025, Wiley-VCH. (G) Thermocouple arrangement in the primary testing region for transient thermal-management analysis [95]. Copyright 2021, Elsevier. (H) Schematic illustration of an optimized TEG system architecture and electrical performance of the TEG system [96]. Copyright 2023, Multidisciplinary Digital Publishing Institute.

### All-day power-generation systems

Integrating PCMs with radiative cooling extends TE generation beyond daylight by combining daytime heat storage with nighttime heat rejection. As illustrated in Fig. 6D, Zhang et al. [84] developed a hybrid photothermal catalytic–TEG–PCM (PTC–TEG–PCM) system in which the TEG recovered low-grade heat during daytime operation, while the PCM released stored latent heat to extend electricity generation after illumination ceased. The system produced output voltages up to 790 mV under high solar irradiation and operated for more than 12 h under actual outdoor conditions. To further enhance nighttime performance, several advanced system architectures have been proposed. Representative approaches include selective absorber–emitter configurations that combine solar heating and radiative cooling to enhance nocturnal TE output [85], temperature-adaptive PCM radiative coolers [86,87], and microstructure-optimized radiative-cooling materials [88]. Building upon these concepts, researchers have recently developed highly integrated all-day energy-harvesting systems that simultaneously exploit photothermal conversion and radiative-cooling processes (Fig. 6E) [12,89]. Hou et al. [12] reported a “triple-mode” film that integrates photothermal conversion, latent-heat storage, and radiative cooling on a single platform. The film exhibited a photothermal conversion efficiency of 93.7%. During daytime operation, solar heating drove TE power generation while charging the PCM with thermal energy. At night, the stored latent heat was released while radiative cooling created a low-temperature region on the film surface, maintaining the temperature gradient required for TE generation. The system achieved a maximum power density of 21.1 W·m^−2^, providing a promising pathway for solar-energy storage and reuse (Fig. 6F). After 200 differential scanning calorimetry cycles, the phase-change temperature and enthalpy remained nearly unchanged, while 50 photothermal cycles produced nearly overlapping temperature-response curves with a peak-temperature fluctuation within ±1.2 °C. More recent systems have extended these strategies through integrated device design and switchable spectral regulation. Wei et al. [90] experimentally investigated a PV–TEG–PCM system incorporating nocturnal sky radiative cooling, reporting TEG voltages of up to 0.29 V during daytime and 0.15 V at night. Kang et al. [91] combined a solar absorber, paraffin PCM, Fresnel lens, and radiative cooling, achieving power densities of 4.1 mW·cm^−2^ during daytime and 0.2 mW·cm^−2^ at night. In a complementary switchable solar-heating/radiative-cooling TE architecture, Wang et al. [92] used a VO_2_ phase-transition layer to regulate the spectral response, with infrared emissivity changing from 0.366 in daytime heating mode to 0.866 in nighttime cooling mode. Together, these studies highlight the need to coordinate solar absorption, latent-heat storage, and radiative heat rejection, while related research addresses dynamic thermal regulation [93] and worldwide radiative-cooling potential [94]. Because the balance depends on spectral properties and environmental conditions, system comparisons should distinguish open-circuit voltage from load-delivered power and evaluate performance over the full day–night cycle.

### Transient thermal-load regulation

In practical applications, heat sources often exhibit substantial temporal fluctuations, leading to unstable TE output and accelerated thermal-fatigue degradation. The incorporation of PCMs as dynamic thermal buffers effectively mitigates heat-flow oscillations, improves thermal robustness, and extends power-generation duration. On the one hand, PCMs transform transient thermal spikes into smoother thermal inputs. As illustrated in Fig. 6G, Huang et al. [95] proposed integrating PCMs into automotive TE systems. Compared with a TEG without PCM, the PCM-integrated configuration increased the average open-circuit voltage and power output by 0.7% and 1.16%, respectively. On the other hand, proper thermophysical matching between PCMs and TEMs is essential for maximizing energy-utilization efficiency. Tian et al. [8] demonstrated through numerical simulations that selecting an appropriate phase-transition temperature enables TE devices to operate within their optimal efficiency range for extended periods. By matching the PCM phase-transition temperature to the TEG’s TE characteristics, the total generated electricity increased by up to 15.6% compared with the corresponding nonoptimized PCM–TEG case. Furthermore, achieving superior transient TE performance requires the synergistic optimization of multiple design parameters. Shi et al. [96] employed the Taguchi method to quantitatively evaluate the effects of PCM layer thickness, PCM type, and heat source power on transient TE performance. As shown in Fig. 6H, the optimized system, consisting of a TEM, a heat sink, and a thermal-storage unit, achieved a maximum voltage of 3.0394 V and a peak power output of 2.3095 W.

Overall, system integration serves as the critical bridge that transforms material-level advantages into practical application performance. Through rational architecture design, transient thermal management, all-day operational strategies, and targeted optimization for high-performance scenarios, PCM–TEG systems are gradually evolving from passive coupling schemes toward actively regulated energy platforms, demonstrating enormous potential for next-generation sustainable energy technologies. Current studies also tend to optimize PCM properties and device parameters separately; a more rigorous strategy is to match phase-transition temperature, latent heat, and total thermal resistance to the fluctuation time scale so that heat-flow smoothing translates into a sustained time-dependent temperature difference and higher time-integrated power output.

## Emerging Application Frontiers: Cross-Disciplinary Expansion of PCM-Enabled STE Systems

Driven by continuous advances in system architecture and multifunctional integration, PCM-enabled STE technologies have expanded beyond conventional power-generation applications toward flexible electronics, multienergy cogeneration systems, deep-sea energy harvesting, and concentrated photovoltaic (CPV) platforms, demonstrating remarkable interdisciplinary potential.

### Flexible wearable electronics and human thermal management

In wearable systems, breathability and mechanical flexibility are critical factors governing user comfort and long-term usability. To address these requirements, numerous multifunctional composite materials have been developed to simultaneously provide energy-harvesting capability and structural robustness [97]. STE films constructed from porous fibrous networks and bioinspired interfaces (Fig. 7A) can achieve broadband solar absorption while maintaining high water-vapor permeability. Such systems continuously power physiological sensors under low-irradiation conditions and generate output voltages up to 1.2 V under 1-sun illumination [98]. To further improve wearing comfort, Miao et al. [99] optimized the device architecture and packaging materials in a wearable TEG, achieving a high-power density of 18.4 μW·cm^−2^ while simultaneously reducing skin thermal resistance and pressure sensation (Fig. 7B). For intelligent thermal regulation, Geng et al. [78] developed an ultraflexible PCM inspired by biological muscle tissues. Through the incorporation of dynamic cross-linked networks, the material exhibited programmable deformation and thermally driven actuation. Remarkably, it was capable of lifting objects weighing more than 2,620 times its own weight while delivering a work density of 1,330 kJ·m^−3^, enabling adaptive thermal management during human motion. Moreover, Meng et al. [100] designed an interface-engineered CPCM that could be assembled into flexible modular arrays. The resulting system generated voltages up to 650 mV under 1-sun irradiation and achieved continuous power generation when integrated with solar-assisted TEMs, highlighting its potential for next-generation wearable electronics (Fig. 7C). However, high laboratory output alone does not guarantee wearable viability; future designs should jointly benchmark power density, skin-side thermal resistance, breathability, bending/sweat durability, and long-term contact stability under realistic human motion.

**Fig. 7.**
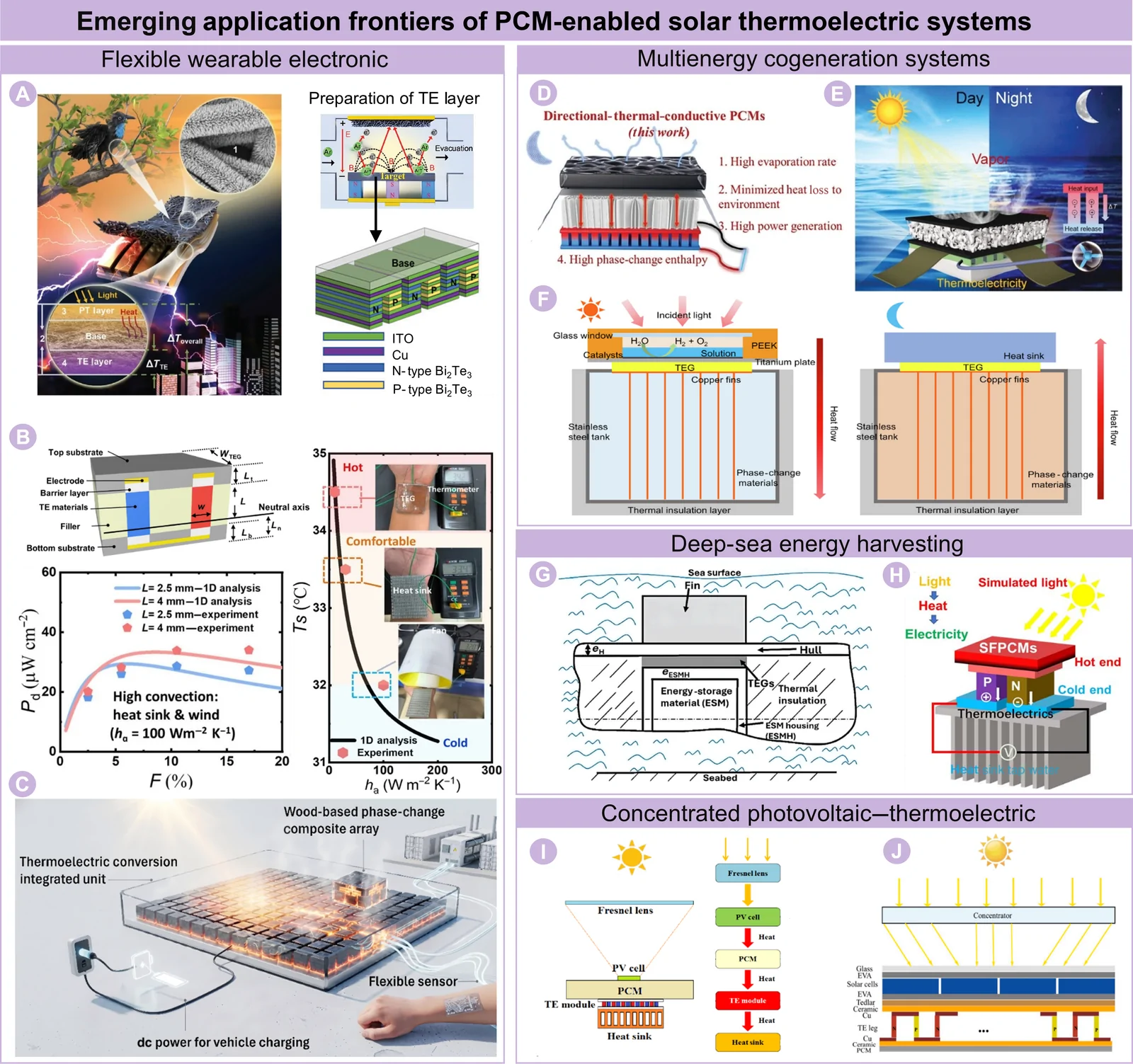
Emerging application frontiers of phase-change material (PCM)-enabled solar–thermal–electric (STE) systems. (A) Schematic illustration of an integrated STE film and the fabrication process of the photothermal layer in a highly flexible and breathable photo-thermal-electric membrane (FB-PTEM) [98]. Copyright 2024, Wiley-VCH. (B) Internal structure of a wearable thermoelectric generator (TEG), together with its heat-transfer behavior under natural convection and thermoelectric (TE) performance under different cooling conditions [99]. Copyright 2024, Springer Nature. (C) Conceptual illustration of the potential applications of TBAWP systems [100]. TBAWP refers to a stearic acid-loaded, interface-engineered wood-based composite PCM incorporating BPNS@MPN, Ag nanoparticles, and grafted octadecyl chains. Copyright 2026, Wiley-VCH. (D) Schematic highlighting the advantages of PCM with directional thermal conductivity [102]. Copyright 2024, Wiley-VCH. (E) Conceptual design of an all-day solar-driven steam-generation and TE-power-generation system [112]. Copyright 2023, Wiley-VCH. (F) Schematic illustration of a photothermal catalytic (PTC)–TEG–PCM platform for simultaneous hydrogen production, electricity generation, and thermal-energy storage under daytime illumination, as well as nighttime power generation driven by latent-heat release from the PCM [84]. Copyright 2024, Wiley-VCH. (G) Conceptual design of a TEG and PCM-assisted autonomous underwater vehicle for deep-sea energy harvesting [113]. Copyright 2018, Elsevier. (H) Experimental setup for STE energy conversion based on shape-stabilized phase-change composites [106]. Copyright 2023, Royal Society of Chemistry. (I) Structural design of the photovoltaic (PV)–PCM–TE hybrid system, together with the corresponding energy-flow pathways and electrical-output comparison [107]. Copyright 2017, Elsevier. (J) Schematic illustration of the concentrated photovoltaic thermal (CPVT)–TE system integrated with PCMs [109]. Copyright 2023, Elsevier.

### Multienergy cogeneration systems

To maximize solar-energy-utilization efficiency, PCM-enabled STE systems are increasingly integrated with interfacial evaporation technologies and PTC processes. Local solar–thermal gradient regulation also strongly affects solar evaporation and water-collection performance [101]. By constructing directional thermal conduction pathways and tailoring interfacial wettability, highly efficient water-to-heat conversion can be achieved while simultaneously directing residual heat toward TEG. Such systems have demonstrated evaporation rates of up to 2.51 kg m^−2^ h^−1^ and power outputs of 1,759.7 mW·m^−2^ (Fig. 7D) [102]. As illustrated in Fig. 7E, PCMs can recover latent heat during condensation or store excess solar energy, which substantially improves the stability of water–electricity cogeneration systems [13,102,103]. A representative example is the PTC–TEG–PCM platform developed by Zhang et al. (Fig. 7F) [84]. In this system, solar energy drives photocatalytic hydrogen production from seawater, while TEMs convert low-grade waste heat into electricity. The PCM stores excess thermal energy and stabilizes the reaction temperature. Compared with a traditional PTC system, the integrated PTC–TEG–PCM configuration increased the overall energy-conversion efficiency by approximately 500% while maintaining the solar-to-hydrogen efficiency. In addition, PCM-mediated reaction-temperature regulation has attracted widespread attention as a means of improving PTC hydrogen-production efficiency [104]. The main limitation is that added energy-conversion functions can compete for the same heat flux and increase system complexity; future systems should therefore optimize energy/exergy allocation among evaporation, catalysis, storage, and TE generation using total useful-energy yield rather than maximizing any single output.

### Deep-sea energy harvesting

Because PCM-enabled systems contain no moving components and require minimal maintenance, they are particularly attractive for extreme environments such as deep-sea applications. Saini and Osorio [105] systematically investigated PCM–TEG systems for harvesting ocean thermal energy (Fig. 7G). In this concept, PCMs serve as thermal-storage media, and repeated phase transitions are induced by the temperature gradient encountered during the ascent and descent of autonomous underwater vehicles. The harvested thermal energy is subsequently converted into electricity, reducing dependence on conventional batteries and extending underwater operational durations. To address challenges associated with PCM shape stability in marine environments, Zhou et al. [106] developed a self-floating STEG incorporating an ultraflexible PCM (Fig. 7H). The system combined mechanical robustness against wind and wave disturbances with efficient STE, achieving output voltages as high as 410 mV and demonstrating considerable potential for solar-energy harvesting in ocean environments. Practical deployment nevertheless remains constrained by hydrostatic pressure, salinity-induced corrosion, encapsulation fatigue, and slow environmental thermal cycling, requiring pressure-tolerant packaging and long-cycle validation based on harvested energy per cycle rather than open-circuit voltage alone.

### CPV–TE hybrid systems

In CPV systems, excessive waste heat [7] and the associated temperature rise of PV cells remain major challenges that limit overall system performance. The integration of PCM–TEG modules provides an effective solution by simultaneously converting waste heat into electricity and regulating the operating temperature of PV cells. Such a strategy not only enables more efficient utilization of the solar spectrum but also enhances the long-term stability and efficiency of CPV systems. As illustrated in Fig. 7I, Cui et al. [107] developed a novel CPV–TE–PCM hybrid system in which infrared waste heat not absorbed by the PV layer was transferred to an underlying PCM reservoir. The configuration effectively cooled the PV layer and improved PV power output while simultaneously supplying thermal energy to the TEG. The configuration, therefore, established a cascaded energy-utilization pathway of “light–electricity–heat–electricity”, substantially enhancing overall solar-energy-utilization efficiency. To address the limited temperature-regulation range and poor long-term adaptability of conventional single-layer PCM cooling systems, Chibani et al. [108] introduced a multilayer PCM cascade-cooling architecture. The proposed design reduced the surface temperature of PV panels to approximately 25 °C and improved electrical conversion efficiency by about 5.18%, demonstrating the effectiveness of hierarchical thermal management. Furthermore, Rejeb et al. [109] established a numerical model of a CPV thermal–TE system (Fig. 7J), confirming that the latent heat stored in PCMs can simultaneously enhance TE power generation and suppress CPV temperature rise under hot-climate conditions. A key unresolved trade-off is that stronger PV cooling can reduce the temperature gradient available to the TEG; future CPV–TE–PCM systems should therefore co-optimize spectral splitting, PCM transition temperature, thermal resistance distribution, and heat-rejection conditions to maximize total system energy rather than the efficiency of an individual component.

In summary, from flexible wearables to combined resource production and to environmental applications such as deep-sea and concentrated photovoltaics, photothermal–phase change coupling technology is continuously expanding its scope of application, demonstrating considerable cross-disciplinary development potential. This also signals the accelerated emergence of a new generation of energy technologies characterized by distributed, adaptive, and multienergy output.

## Conclusions and Outlook

This review summarizes advances in PCM-buffered STE conversion across material design, CPCM architectures, and system integration. Photothermal absorbers, conductive fillers, confinement strategies, and engineered interfaces improve solar absorption, thermal transport, storage, and stability, while PCM–TEG coupling prolongs electricity generation. Challenges include interfacial resistance, cycling degradation, limited adaptability, and system complexity. As Fig. 8 illustrates, future directions include molecular-scale interfacial engineering, adaptive phase-change regulation, artificial-intelligence (AI)-assisted multiscale modeling of coupled optical–thermal–phase-change-TE processes, and integration with photovoltaics, batteries, and heat pumps for electricity–heat–chemical cogeneration. In this context, several key issues need to be addressed: (a) standardized evaluation reporting irradiation intensity/duration, device dimensions, ambient/cold-side conditions, electrical loading, transient temperature differences, power density, operating duration, and integrated electrical energy; (b) reliability assessment combining cycle counts with retention of latent heat, thermal conductivity, photothermal efficiency, electrical output, and interfacial integrity; (c) techno-economic and life-cycle assessments covering fabrication costs, manufacturing complexity, service lifetime, maintenance, embodied energy, emissions, and recyclability; and (d) experimentally validated physics-informed/AI-assisted datasets incorporating optical and temperature-dependent thermophysical properties, phase transitions, interfacial resistance, geometry, irradiation history, thermal boundaries, and electrical loads, with predictions verified against transient temperatures, TEG temperature differences, voltage/current, power, and integrated electrical energy. Addressing these challenges is essential for translating PCM-buffered STE technologies from laboratory research into practical applications under diverse operating conditions.

**Fig. 8.**
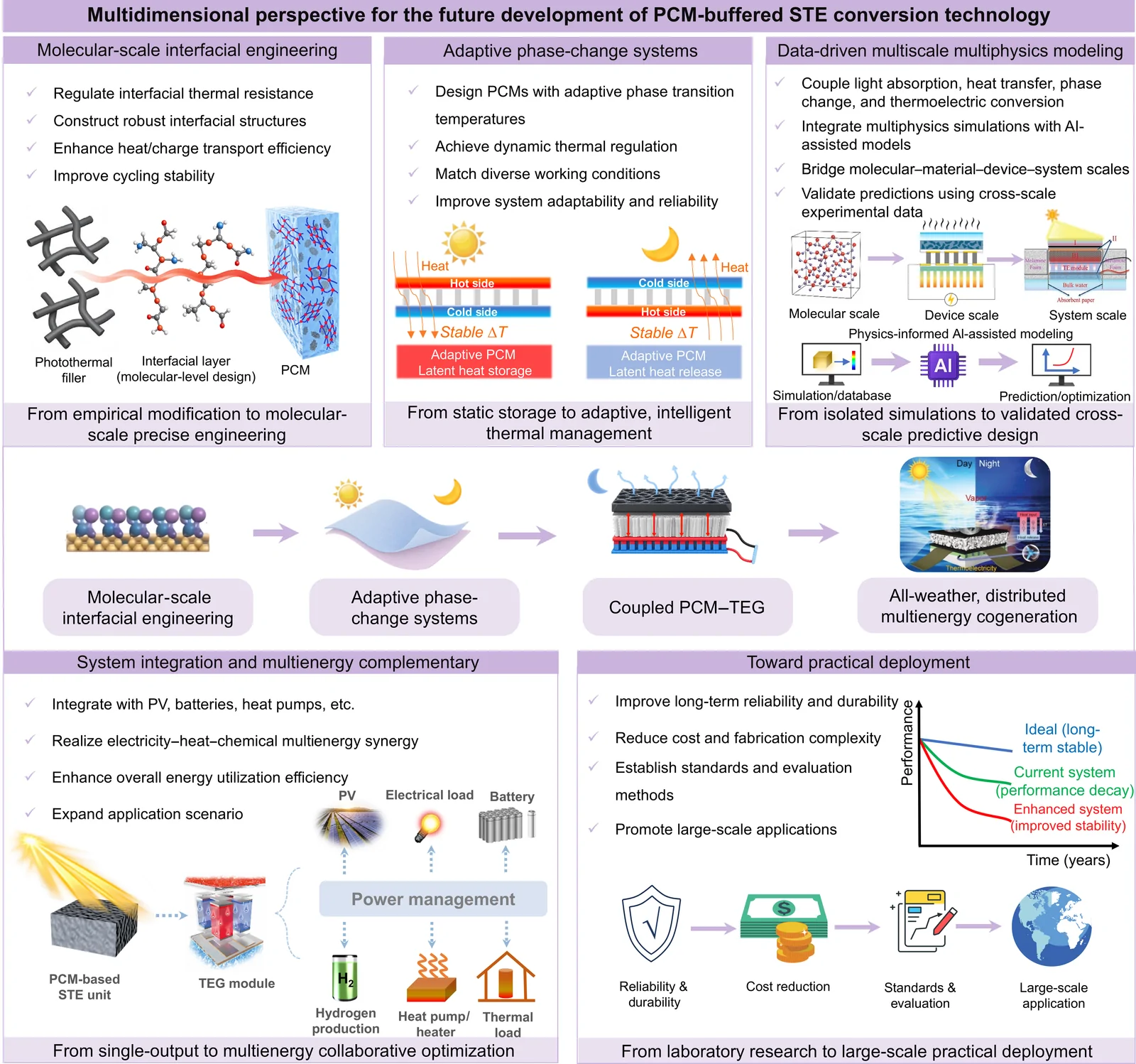
Multidimensional perspectives on the future development of phase-change material (PCM)-buffered solar–thermal–electric (STE) conversion technologies, including molecular-scale interfacial engineering, adaptive phase-change systems, data-driven multiscale multiphysics modeling, and multienergy system integration, from material and interface optimization to reliable, cost-effective, and large-scale practical deployment [31,78,102,111,112,114]. Copyright 2024, Wiley-VCH; 2025, Elsevier; 2024, Wiley-VCH; 2025, Wiley-VCH; 2023, Wiley-VCH; and 2026, Wiley-VCH.
